# A comparative case study of prescribing and non-prescribing physiotherapists and podiatrists

**DOI:** 10.1186/s12913-020-05918-8

**Published:** 2020-11-24

**Authors:** Nicola Carey, Judith Edwards, Simon Otter, Heather Gage, Peter Williams, Molly Courtenay, Ann Moore, Karen Stenner

**Affiliations:** 1 School of Health Sciences, University of Surrey, Kate Granger Building, Guildford, GU2 7YH UK; 2 School of Health Science, University of Brighton, Brighton, UK; 3 Department of Clinical and Experimental Medicine, Surrey Health Economics, University of Surrey, Guildford, UK; 4 Department of Mathematics, Faculty of Engineering and Physical Sciences, University of Surrey, Guildford, UK; 5 School of Healthcare Sciences, Cardiff University, Cardiff, UK

**Keywords:** Physiotherapy, Podiatry, Independent prescribing, Comparative case study

## Abstract

**Background:**

Increasing numbers of nurses, pharmacists and allied health professionals across the world have prescribing rights for medicines: over 90,000 of the eligible United Kingdom workforce are qualified as non-doctor prescribers. In order to inform future developments, it is important to understand the benefits and impact of prescribing by allied health professionals including physiotherapists and podiatrists.

**Aim:**

to compare outcomes of physiotherapist and podiatrist Independent Prescriber (PP- IP) patients with those of physiotherapist and podiatrist non-prescribers (PP-NPs). Outcome measures included patient satisfaction, ease of access to services, quality of life and cost implications.

Design: a mixed method comparative case study.

**Methods:**

Using mixed methods of data collection, outcomes were compared between 7 sites where care was provided from a PP-IP (3 podiatrist and 4 physiotherapist IPs) and 7 sites from a PP-NP (3 podiatrist and 4 physiotherapist NPs). Patients were followed up for 2 months (2015–2016).

**Results:**

489 patients were recruited: *n* = 243 IP sites, and *n* = 246 NP sites. Independent prescribing was found to be highly acceptable, and equivalent in terms of quality of life (*p* > 0.05) and patient satisfaction (*p* ≤ 0.05) compared to care provided by NPs. PP-IP care delivery was found to be more resource intensive than PP-NP, with longer consultation duration for IPs (around 6.5 mins), and a higher proportion of physiotherapy patients discussed with medical colleagues (around 9.5 min).

**Conclusion:**

This study provides new knowledge that PP-IPs provide high levels of care. PP-IP care delivery was found to be more resource intensive. Further research is required to explore cost effectiveness. A more focussed exploration within each profession using targeted outcome measures would enable a more robust comparison, inform future developments around the world and help ensure non-doctor prescribing is recognised as an effective way to alleviate shortfalls in the global workforce.

## Background

As life expectancy increases, and the world’s population continues to grow [1–3], many countries are shifting the focus of their health system from acute to chronic diseases, alongside managing increasing service demands [4]. Recent data from the United Kingdom (UK), United States (US) and across Europe confirms 25% of adults take three or more medicines each day [2, 5] and that by 2020 the world’s population will receive 4.5 trillion doses of medicine each year [5–7].

There is however, a worldwide deficit of 18 million health workers [8], with a predicted 350,000 shortfall in the UK, and a third of the current workforce due to retire by 2030 [9]. Inadequacies with traditional doctor/physician-led care systems mean that in order to maintain patient access to prescription medicines, new approaches are imperative [9, 10]. Allied Health Professionals, (health professionals who are not medical doctors, physicians, nurses. Pharmacists or dentists), e.g. therapeutic radiographers, paramedics, podiatrists and physiotherapists (AHP) have in particular been identified as having an integral role to the required transformational change [11].

Extending prescribing rights to nurses, pharmacists and allied health professions [12, 13] has been the focus of a UK policy drive to improve services and access to medicines by making better use of existing skills and support service innovation [11, 14–16]. Of the 907,000 UK healthcare professionals entitled to undertake prescribing training [17], over 90,000 of the eligible workforce are now qualified as prescribers [17], placing the UK as the forerunner in the development of non-doctor prescribing, also known as non-medical prescribing, worldwide.

In the UK, Independent Prescribing (IP) and Supplementary Prescribing (SP) are two different forms of non-doctor prescribing. Training typically involves 27 classroom days, a mandatory aspect of supervised practice, and robust academic and practice assessment [18–20], a dual qualification in IP and SP being awarded to registered nurses, pharmacists, radiographers and paramedics, podiatrists and physiotherapists. Supplementary prescribing rights were extended to some allied health professions in 2005, with further changes to legislation in 2013 permitting physiotherapists and podiatrists to prescribe medicines independently [21–23]. Apart from some restrictions around independent prescribing of controlled drugs and in line with other allied health professions, physiotherapists and podiatrists, normally with 3 years relevant post qualification experience, are able to independently prescribe any medicine within their area of competence without the need for a doctor. By contrast supplementary prescribing defined as dependent prescribing, is based on an initial diagnosis by a doctor and an agreed clinical management plan detailing medicines that can be prescribed [24].

Although several other countries, including Australia, Ireland, and Netherlands, have seen similar developments in non-medical prescribing, approaches to training, accreditation and models of prescribing practice are varied [25–28]. Physiotherapists have for example, authorisation to provide advice about and/or to administer or supply medicines in some states in Australia, New Zealand and Canada, but only those in the US military can prescribe [29, 30]. Podiatrists have similar authority in Australia and some European countries but are only entitled to prescribe in some Canadian provinces [29, 31].

When used by nurses and pharmacist, independent and supplementary prescribing are reported as acceptable and beneficial to patients, with some evidence of enhanced clinical outcomes compared to doctors [26, 32–34]. A recent systematic review reported that non-medical prescribing has no adverse impact upon patient outcomes, patient satisfaction or resource utilisation [35]. Reviews on the impact of extended physiotherapist roles reveal research hampered by small numbers, role variation and poor role definition [36, 37], literature dominated by service descriptions and audit with positive reporting bias [29, 36, 37], and a lack of evidence regarding podiatric practice [29]. Whilst physiotherapist and podiatrist supplementary prescribing helps streamline service delivery [38, 39], independent prescribing is expected to bring additional benefits in line with nurse and pharmacist prescribing [40, 41]. Exploration of clinical and cost effectiveness in this area is limited with inconclusive findings [42–47]. As most evidence relates to nurses and pharmacists, it is important to evaluate the impact of prescribing by allied health professionals in order to inform commissioning and implementation of non-medical prescribing services where they are beneficial.

Seven years after the introduction of current legislation enabling physiotherapists and podiatrists to independently prescribe medicines, there were (as of October 2020) 1295 physiotherapists and 442 podiatrists with an annotation as independent prescriber, with a further 108 physiotherapists and 67 podiatrists registered as supplementary prescribers [48]. There is a lack of evidence of reporting on physiotherapist and podiatrist independent prescribing practice, or the medicines they prescribe and no studies available which quantify the impact of podiatrist and physiotherapist independent prescribing on patient satisfaction, access to services, quality of life or report cost-implications of care delivery [29]. This is important given the increasing emphasis in the UK and around the world on extending prescribing rights to nurses, pharmacists and allied health professionals as a key strategy in addressing workforce deficits and ensuring patients have ongoing access to medicines [8–10, 49].

## Methods

### Aim

Was to compare the outcomes of patients managed by physiotherapist and podiatrist independent prescribers (PP- IP) with those under the care of physiotherapist and podiatrist non-prescribers (PP-NPs). Outcome measures included patient satisfaction, ease of access to services, quality of life and costs.

#### Study design

This study was commissioned to undertake concurrent evaluation of physiotherapist and podiatrist independent prescribing reflecting the 2013 regulatory changes introduced to both professions. A comparative case study methodology used in situations when no single outcome measure is available was adopted [50, 51]. Outcomes were compared between 7 sites where patients received care from a PP-IP (3 podiatrist and 4 physiotherapist IPs) and 7 sites where care was provided by a PP-NP without a prescribing qualification (3 podiatrist and 4 physiotherapist non-prescribers) [52]. Mixed methods (including interviews, structured observation of consultations, patient questionnaires) were used to collect data at each of the 14 sites during a 5-day period of observation of practice. Details of data collection tools, methods and piloting are shown in Table 1.

**Table 1:** Summary of data collection arrangements and instruments

Category of data	Method of data collection	Timing of collection	Items and instruments	Piloting
**1) Characteristics of PPs & sites**	i) Structured interview and site visit	Prior to observation period	Setting and geographic location *PPs profile*: Age, gender, highest educational qualification, salary/band, full/part time status, job title/role; *Service Information*: service description & patient profile, single or multi-professional team, other NMPs in team	Interview schedules were reviewed by research team and project advisory group. Main interviewer (JE) was buddied by experienced team member (KS) for first two interviews in order to provide guidance and clarify and address any issues with the interview schedule. Following this, minor revisions were made to improve the flow of questions.
**2) Patient characteristics**	i) Patient questionnaire 1 & 2	Post consultation and 2 months following	*Socio-demographics*: age, gender, living arrangements; accommodation, employment; education; ethnicity	**Q1***:* Patients (n = 5) completed and commented on ease of comprehension, length and time. Based on comments no refinements were made. **Q2**: piloted concurrently at first site (case-site 3). After first 10 completed, ease of use, consistency and question completion rate were discussed with no amendments or changes required.
**3) Patient reported outcomes**	i) Patient questionnaire 1 ii) Patient questionnaire1 and 2	Post consultation Post- consultation and 2 months following consultation (excluding 1st four sites)	*Patient satisfaction:* with consultation, advice and medicines information comprised subscales from several validated tools (total 24 items): i) *Consultation Satisfaction Questionnaire* i.e. ‘professional care’, ‘perceived time’ and ‘overall satisfaction’ and ii) *Medical Interview Satisfaction Survey (MISS)* [53, 54] ‘compliance intent’ (10 items) & for patients who received medicines information or advice questions from PP iii) *Satisfaction with Information about Medicines (SIMS) Scale* [55] e.g. dose schedule, how medicine works, side-effects, and medicines adherence) [54–57] (14 items). *Service Satisfaction:* was measured by 7 items on ease of access to services from the outpatients’ opinion of quality of hospital departments questionnaire [58]–7 items. *Attitude*s *towards PP-IP* [56, 59]–4 items. 6 point Likert scales (strongly disagree to strongly agree) used for all items Quality of life validated EQ-5D**-**5L [60] comprising 5 dimensions, from independent – dependent, with 5 weighted levels affording a single index value score. (i.e. mobility, self-care, usual activities, pain/discomfort and anxiety/depression)	Formal piloting was undertaken in January 2015 in a secondary care based rheumatology outpatient clinic (not designated as a site). Five completed questionnaires were returned with comments indicating that content, layout and design was comprehensive and completion time was of acceptable length, ranging from 9 to 15 min. Questionnaire 2 was implemented following data collection completion at the first four sites, and was piloted concurrently at the first site visited (site 3) after its approval. After the first 10 completed questionnaires, ease of use, consistency and question completion rate were discussed at team meetings; no amendments or changes were required.
**4) PPs activities**	i) Observation diary completed by researcher ii) Prescriptions	Real-time service delivery up to 5 working days (37 h) Real-time service delivery up to 5 working days (37 h)	Using a Microsoft Access© custom built electronic diary based on previous validated tools [61–63], a researcher recorded details of the model of service provision and MMA (including outcome and prescribing actions) during each observed consultation. *Model of service provision:* Consultation duration (in minutes); type of consultation (face to face, telephone, email) and appointment (initial, follow-up, emergency), service & referral source (e.g. NHS in/outpatient, community, GP, social enterprise, private). Other work activities in relation to care included referrals made (to whom and how), discussion with colleagues, time spent in discussions with colleagues and review arrangements. *MMA* i) *outcome*: whether a new medication was required; decisions to alter, stop, or make no change to existing medications; or decision to repeat prescribe previous item(s); ii) *prescribing actions*; decision to recommend OTC product; recommend to Dr., other prescriber or via hospital notes prescription is required; adjust dose/drug according to pre-agreed protocols; (i.e. PGD; PSD, exemptions); whether provided advice to patients about medicines (i.e. how it works, when to take and side-effects); medication details (i.e. name, dose, duration, formulation) Questions were fixed option and/or free text. All prescriptions issued by PP-IPs during observed consultations were collected and assessed based on previous work [64–66] and guidelines for prescription writing in the BNF [67] (i.e. accuracy, legibility, correct use of terminology, whether medicines were prescribed generically, preparation details, dose, dose frequency, length of treatment, and instructions regarding frequency, location and application of topical treatments).	Details of 8 observed consultations were recorded and downloaded into Microsoft Excel©. Data were found to be comprehensive, and the template layout/design revised following team discussion data. There was no piloting of the assessment tool as it had been used in previous studies [41, 68].
**5) Resource implications and costs**	i) Interviews with PP	Prior to baseline data collection	Grade/ banding of each of the PPs in the study.	(as reported above)
	ii) Observation diary completed by researcher	Real-time service delivery up to 5 working days (37 h)	Six items related to consultations with individual patients were also examined for differences between PP-IPs and NP-PP-NPs -number and duration of consultations - frequency & duration of discussions with colleague or other professional regarding patient’s medication -frequency of new medications -frequency of referrals and follow-up consultations	(as reported above)
	iv) Patient record audit	Clinical records 2 months following consultation. A maximum of 15 patients per site were selected.	Requested investigations, tests (e.g. BP, bloods, x-ray, MRI scan, CT, urine, sputum etc.) and referrals and services used relevant to the presenting complaint (i.e. case site PP, consultant specialist, clinical nurse specialist, GP, GP based nurse/ nurse practitioner, community nurse pharmacists, social services, other healthcare professionals) other hospital outpatients, hospital admissions, and number of in-patient days, A&E visits etc.	Audit tool: was piloted on 8 sets of medical records. Concerns were raised about quality of available data and that retrospective data collection could present difficulties with potential incomplete data. Following data collection at first four sites an amendment to the study protocol, as previously described was made.
	iii) Patient questionnaire 2	2 months following consultation (excluding first 4 sites)	Self- report use of health services for PP related issues in the previous 2 months including: tests received, referrals, follow-up consultations, un-planned consultation; visits or contact with GPs, clinical nurse specialist, pharmacists, social services, other healthcare professionals, hospital outpatient clinics, A&E visits, hospital admissions, and number of inpatient days	

The original intention was to collect data on patient follow-up treatments and re-consultations by audit of clinic records at 2 months. It was evident that this was limited and inconsistent during data collection at the first four sites. The protocol was therefore amended to include a second patient questionnaire for self-report usage of health services in the 2 months after the index consultation. Data collection took place simultaneously January 2015–March 2016.

### Sample size

Anticipating patient satisfaction and ease of access to services being best expressed as positive or negative responses, in order to detect an absolute underlying difference of 40% between PP-IP and NP-PP, with size = 5% and power = 80%, a minimum of 24 subjects were needed in each PP-IP and NP-PP site. Allowing for a dropout rate of 20%, to enable a statistically sound comparison to be made between any specific pair of PP-IP and NP-PP sites, a target recruitment of 30 patients per site (total *n* = 420), collected over a maximum of 5 working days, was set.

Initial sample estimates, based on information provided by physiotherapists and podiatrists in clinical practice, indicated that full-time PP-IPs/NP-PPs have up to 60 consultations, lasting approximately 20–40 min each, per week, generating data on potentially 840 patient care episodes across 14 sites, indicated that, even allowing for repeat patient visits and inclusion criteria failures, such a recruitment was feasible.

#### Case sites

Sites with physiotherapist and podiatrist independent prescribers were purposively selected from an earlier study phase [52] to include diversity with respect to care setting, geographical location and patient demographics across England.

### Recruitment

#### Podiatrists and physiotherapists

Initial email/ telephone contact was made with physiotherapist and podiatrist independent prescribers who had completed an earlier survey whilst undertaking independent prescribing training (*n* = 70) and indicated willingness to participate in further research [52]. Those who expressed an interest were provided with a participant information sheets and supplementary information on case site involvement and requested to ensure organisational and local Research and Development support.

Non-prescribing physiotherapist and podiatrist sites, matched on professional role, care setting, geographical location and using NHS Agenda for Change (Afc) national pay scale banding [69], were either nominated by PP-IPs, identified through personal contacts of the project advisory group or enquiries from individual Research and Development departments via the National Institute of Health Research (NIHR) portfolio. These matched NP-PPs were, with consent, contacted by a member of the research team and recruited following the same process as for PP-IPs. Written informed consent was taken from PP-IPs and NP-PPs on the first day of each case site visit by JE, who assured on-going consent with each PP-IP or NP- PP at the beginning of each contact day.

#### Patients

At each case site a consecutive sample of patients who had scheduled appointments with physiotherapist and podiatrist independent prescribers/ non-prescribers providing adult services during a 5-day (up to 37 h) site visit by the study researcher (JE) were recruited in NHS sites by trained research nurses, and private sites by a second study researcher (EK) between March 2015 and February 2016. Informed written consent was obtained from those who were willing to participate.

A screening log of all patients approached for participation in the study (*n* = 563) was recorded; both those recruited to the study (*n* = 488, 86.7%) and those declining participation (*n* = 75,13.3%), including hospital/unit medical record numbers, gender and the date of consent, by the local research nurse/ study researcher. Following the observed consultation (see Table 1) those who agreed to participate completed and posted Patient Questionnaire 1 into a box in the clinic area or returned using pre-paid envelopes.

#### Data collection

An initial telephone interview, informed by previous work in the area [70] was conducted with the physiotherapist and podiatrist from each site using semi-structured questions to gather information on site characteristics, and professional role. Details of the data collection and instruments, informed by the study patient and public involvement and advisory groups, are presented in Table 1. All data collection instruments were piloted in a non-study physiotherapist independent prescriber NHS outpatient clinic in January 2015, with only minor corrections to wording required (see Table 1).

#### Outcome measures

##### Baseline questionnaire 1

Informed by previous work [70] and several validated tools [53–57, 71, 72] a patient questionnaire was constructed to ensure that the generic questionnaire developed to evaluate prescribing by nurses and midwives in the Republic of Ireland [56] was appropriately adapted.

*Section 1* recorded patient satisfaction with services received at the time of consultation using 10 medical interview satisfaction questions [55, 56] and ‘ease of access’ to services using 7 additional questions [58].

*Section 2* comprised 4 statements measuring patients’ attitudes to physiotherapist and podiatrist independent prescribers (65, 68) and 14 statements about the advice/information they may have received from physiotherapist and podiatrist independent prescribers/ non-prescribers during the consultation including side effects, action of use and dose schedule and medicines adherence [54–57].

*Section 3* employed the validated EQ-5D-5L quality of life profile measure of five dimensions (mobility, self-care, usual activities, pain, anxiety/ depression) rated on five levels (no problem to severe problem/ unable questionnaire [60]. Although the standardized extended EQ-5D incorporates a vertical 20 cm visual analogue scale (VAS) rating scale, patient and public involvement group members consistently reported difficulty indicating numerical values for how they felt at any one time point. It was therefore decided to exclude this from the questionnaire.

*Section 4* comprised 7 items related to general demographics in order to describe respondent characteristics including age, living arrangements, employment, ethnic group and educational attainment.

##### Follow up questionnaire 2

Comprised of 5 questions relating to health resource use in addition to a second completion of the EQ-5D-5L asked over telephone. Patients were asked if they had, in the 2-month period following consultation received medicines prescribed/recommended by the physiotherapist and podiatrist independent prescribers/ non-prescribers, undergone diagnostic tests (e.g. radiology, blood tests), returned to the physiotherapist and podiatrist independent prescribers/ non-prescribers for follow-up treatment, been referred to other services/professionals, or received unplanned treatment for the same condition following the initial consultation (list of 10 potential services) (see Table 1).

#### Data analysis

Quantitative data were entered on to SPSS© Version 22. Descriptive statistics were used to summarise the data and reported where open text data (specifically in relation to medication details and requested tests from the observation diary) had been converted to numeric data. Patient satisfaction and ease of access to services were measured on a 5-point Likert scale or as Yes/No responses. The Likert scale responses were easily reducible to positive or negative responses.

When assessing change in EQ-5D-5L descriptive health score from Patient Questionnaire 1 to Questionnaire 2, a paired t-test was used.

When comparing 2 subgroups for normally distributed outcomes (notably change scores from Questionnaire 1 to Questionnaire 2, such as for overall EQ-5D-5L score), an unpaired t-test was utilised.

When comparing 2 subgroups (in particular prescribing and non-prescribing) for an ordinal outcome, a Mann-Whitney U test was utilised. When comparing 2 subgroups (notably Podiatry and Physiotherapy or prescribing and non-prescribing) for a categorical outcome, the Chi-Squared test was used, reverting to a Fisher’s Exact test in 2 × 2 cross tabulations if 1 or more expected cell count was found to be < 5.

#### Economic analysis

Seven resource implications of independent prescribers compared to non-prescribers were originally considered: rates of prescribing tests ordered; referrals to other health professionals; frequency of follow up; consultation duration; time spent discussing the patient with other colleagues; unplanned consultations for the same condition within two months of the index consultation. Data were gathered through the observation diary, except for tests (from the retrospective audit) and unplanned consultations (from the patient follow up questionnaire). Group level comparisons of independent prescribers compared to non-prescribers for physiotherapists and podiatrists were undertaken separately for each of the seven variables.

The cost implications (British pounds 2015) of differences in consultation length and colleague’s time spent in discussion were examined by applying nationally valid unit costs [73]. A comprehensive micro level costing analysis could not be conducted because data on tests and unplanned consultations were only gathered for a sample of patients and insufficient details were available on medications, referrals and planned follow up to enable costs to be reliably ascribed. Costs that could be estimated were considered in relation to outcomes (satisfaction with consultation, satisfaction with advice, changes in health-related quality of life (EQ-5D-5L) between baseline and follow up) in a simple cost consequences framework.

## Results

### Characteristics of participants

i) *PPs and case sites*

Seven matched pairs of sites, (3 podiatry and 4 physiotherapy) were recruited. Sites were based across 8 Academic Health Science Networks in England (https://www.ahsnnetwork.com/), provided adult services, a mixed range of settings, including private practice (*n* = 2), primary care (*n* = 6), secondary care (n = 6), social enterprise (n = 2) and were well matched by professional role, care setting and agenda for change banding **(see** Table 2**).** All physiotherapist and podiatrist independent prescribers had been qualified for at least 12 months prior to data collection. A total of 488 patients were recruited: 243 across the PP-IP sites with 245 across the NP-PP sites.

**Table 2:** Characteristics of the sites and Physiotherapists and Podiatrists

Pair	Case study site	No. Patients Recruited	Type of PP	Job Title	Setting	Location in England *	Age	Salary band	Full or part time < 30 h in practice	Education highest	Single or multi-professional team	Patient questionnaire 1	Follow up- Patient Questionnaire2	Prescriptions
1	1	49	PO-IP	General/Private	Private	London	71	8a	Full time	Doctorate	single	40	N/A	0
	2	46	PO-NP	General/Private	Private	London	47	12	Full time	Masters	single	35	N/A	n/a
2	3	33	PO-IP	Specialist	Secondary care, NHS In/outpatient	Wessex	41	7	Full time	Masters	multi-professional	22	19	6
	8	37	PO-NP	Specialist	NHS primary & secondary (& private)	Kent, Surrey, Sussex	39	6	Full-time	Degree	single	25	22	n/a
3	10	51	PO-IP	Surgeon/consultant	NHS secondary (& private)	Oxford	59	9	Full time	Masters	multi-professional	32	38	3
	6	42	PO-NP	Surgeon/consultant	NHS secondary	North East & North Cumbria	47	9	Part-time	Masters	multi-professional	26	23	n/a
4	7	6	PT-IP	Specialist	Community	London	31	7	Part-time	Masters	multi-professional	25	N/A	0
	4	11	PT-NP	Specialist	NHS Primary, Community care	Kent, Surrey, Sussex	47	8a	Full time	Masters	multi-professional	25	N/A	n/a
5	9	42	PT-IP	Specialist	Primary, community Social enterprise	Kent, Surrey, Sussex	46	8a	Full time	Diploma	multi-professional	2	2	3
	5	38	PT-NP	Surgeon/consultant	Tier 2 NHS ESP assessment service	Wessex	42	8a	Part-time	Doctorate	multi-professional	6	3	n/a
6	11	41	PT-IP	Specialist	Acute Foundation Trust	Northwest coast	58	8a	Full time	Masters	multi-professional	27	29	0
	12	35	PT-NP	Surgeon/consultant	NHS secondary care	Kent, Surrey, Sussex	48	8c	Full time	Masters	multi-professional	19	23	n/a
7	13	21	PT-IP	Specialist	NHS primary & community Social enterprise	Kent, Surrey, Sussex	52	8a	Full time	Masters	multi-professional	8	16	3
	14	36	PT-NP	Specialist	Primary & community Social enterprise	Kent, Surrey, Sussex	38	8a	Full time	Masters	multi-professional	23	20	n/a
**Totals**		**488**										**315**	**195**	**15**

Job title**: Surgeon/Consultant** (consultant physiotherapists, consultant podiatric surgeons**) General/Private** (physiotherapists practitioners, physiotherapists, podiatrists)

**Specialist** (e.g. Clinical specialist physiotherapists, extended scope physiotherapist clinical specialist podiatrists, clinical lead or senior podiatrists)

Nearly all consultations (*n* = 474), both independent prescribers and to non-prescribers, were face to face (*n* = 473, 99.8%), duration 2–203 min. There was considerable variation in the location of services: 39.2% (*n* = 186) of consultations were provided in NHS hospital outpatients, 25.1% (*n* = 119) NHS community clinics, 20.3% (*n* = 96) private practice, 9.7% (*n* = 46) general practice, 4.4% (*n* = 21) social enterprise and 1.3% (*n* = 6) community service. Of the observed consultations 112 (23.6%) included a medicine related activity, where either a new medication, repeat medication (same dosage) or repeat medication with a change to dosage was required, with patients requiring a total of 124 items of medicine (see Table 3).

**Table 3:** Consultations with medicines related activity

Number of observed consultations	Physiotherapist IP	Number of items	Physiotherapist NP	Number of items	Podiatrist IP	Number of items	Podiatrist NP	Number of items
	***n*** = 107	***n*** = 37	***n*** = 115	***n*** = 29	***n*** = 128	***n*** = 45	***n*** = 124	***n*** = 13
Consultations with no medicines related activity	75	n/a	87	n/a	93	n/a	114	
*Consultations with medicines activity*								
New medication	23	21 × 1 2 × 2 3 × 1	27	26 × 1 1 × 2	31	27 × 1 3 × 2 1 × 4	10	7 × 1 3 × 2
Repeat medication (same dosage)	9	9 × 1	1	1	2	2 × 2	0	0
Repeat medication (dosage changed)	0	0	0	0	2	2 × 2	0	0
Total number of consultations with medicines related activity	***n*** **= 32**	n/a	*n* = 28	n/a	*n* = 35	n/a	n = 10	n/a

Almost all medicines related activity within physiotherapy sites, both independent prescribers and non-prescribers, was related to pain and movement control, either via pain medication or through injection therapy. There was one incident where a patient was advised to alter contraception use following surgery by an independent prescriber. A wider range of medication types were used by podiatrists, both independent prescribers and to non-prescribers, the most common being anti-microbial/anti-fungal topical creams, antibiotics and pain medication. Patients requiring medicines recommended by non-prescribers, both podiatrists and physiotherapists, were subsequently referred to a medical doctor in the usual way.

ii) *Patients*

Demographic data (see Table 4) were collected from 315/ 468 (67.3%) patients who consented to and returned patient questionnaire 1: 49.5% (*n* = 156) were from prescribing and 50.5% (*n* = 159) from non-prescribing sites. A lack of benchmark data with which to compare the patient data means it is not possible to confirm how representative our sample is with respect to the larger population. However, the samples, from the prescribing and non-prescribing group in this study were similar in terms of age, employment status, level of formal education, and ethnic group (*p* > 0.05).

**Table 4:** Patient characteristics

	Physiotherapy n (%)	Podiatry n (%)	Total n = number of responses	% of total sample
**Professional group**				
Which professional consulted	135 (42.86%)	180 (57.14%)	315	100%
**Gender**			*n* = 254	
Male	34 (30.4%)	55 (38.7%)	89	35%
Female	78 (69.6%)	87 (61.3%)	165	65%
**Age**				
Physiotherapy group: *n* = 111, mean 59.7, SD 16.6, (range 17.6–100.98)				
Podiatry group: *n* = 139, mean 67.1, SD 16.16, (range 16.17–94.32)				
Total: *n* = 250, mean 63.8, SD 16.7				
**Living arrangements**			*n* = 257	
Live alone	19 (17.4%)	32 (21.6%)	51	19.8%
Live with other adult(s)	90 (82.6%)	94 (63.5)	184	71.6%
Care home resident	0	22 (14.9%)	22	8.6%
**Type of accommodation**			*n* = 276	
Owner occupied house/flat	97 (82.2%)	104 (65.8%)	201	72.8%
Privately rented house/flat	12 (1.02%)	12 (7.6%)	24	8.7%
Local authority/housing association/cooperative	9 (7.6%)	13 (8.2%)	22	8%
Residential or care home, hospice	0	29 (18.4%)	29	1.05%
**Employment group**			*n* = 262	
In paid or voluntary employment	46 (41.1%)	40 (26.7%)	86	32.8%
Unemployed/student/at home/sick	15 (13.4%)	12 (8%)	27	10.3%
Retired	51 (45.5%)	98 (65.3%)	149	56.9%
**Educated beyond 18 years**			*n* = 274	
Yes	32 (27.4%)	51 (32.5%)	83	30.3%
No	85 (72.6%)	106 (67.5%)	191	69.7%
**Ethnic group**			*n* = 283	
White	117 (96.7%)	160 (98.8%)	277	97.9%
Other	4 (3.3%)	2 (1.2%)	6	2.1%

#### Follow up questionnaire 2

A response rate of 73.7% (197/267) was obtained for questionnaire 2. This sample excluded the 175 participants from the first 4 sites (Sites 1, 2, 4, 7) (see Table 2). Of the remaining 313 participants, 285 consented to follow-up, however contact details were incorrect or missing for 18 participants, leaving 267 eligible to participate.

iii) *Patient outcomes*
  - *Satisfaction and access to services*

The majority of patients (75.9%, *n* = 239) agreed that physiotherapists and podiatrists should be able to prescribe medicines for patients, however 23.2% (*n* = 73) would prefer a doctor to prescribe. Levels of satisfaction for the sample as a total were high, with over 60% positive agreement on all items other than ability to contact the service in an emergency (*n* = 144, 44.4%). Satisfaction with 17 specified aspects of the consultation and services provided by physiotherapists and podiatrists indicated a significantly higher level of satisfaction among the patients of physiotherapist and podiatrist independent prescribers than those of non-prescribers in 8 instances (Table 5).

**Table 5:** Patient views and experience of satisfaction with care received from physiotherapist or podiatrist

Patient views and experience of consultation with physiotherapist or podiatrist (R) indicates reverse score item	Physiotherapist Independent Prescriber (***n*** = 62)	Physiotherapist Non-prescriber (n = 73)			Mann- Whitney U-test	Podiatrist Independent Prescriber (***n*** = 94)	Podiatrist Non-prescriber (***n*** = 86)				Total ***n*** = 315	
	Strongly Agree/Agree (compared with strongly disagree/disagree/no opinion)					Strongly Agree/Agree (compared with strongly disagree/disagree/no opinion)					Strongly Agree/Agree	
	n	% sample	n	% sample	p*	n	% Sample	n	% sample	P *	n	%
1. Overall I was satisfied with the consultation from this physiotherapist or podiatrist	59	95.1%	67	91.2%	0.280	85	90.4%	80	93.0%	0.281	291	92.4%
2.The physiotherapist or podiatrist was very careful to check everything when carrying out my care	60	96.8%	69	94.5%	0.092	82	87.2%	77	89.5%	0.367	288	91.4%
3.I will follow the advice of this physiotherapist or podiatrist because I think she/he is right	59	95.1%	64	87.7%	**0.021**	81	86.2%	75	87.2%	**0.020**	279	88.6%
4.The time I was able to spend with the physiotherapist or podiatrist was a bit too short (R)	46	74.2%	61	83.6%	0.807	68	81.0%	59	68.6%	0.333	234	74.3%
5.The physiotherapist or podiatrist explained the reasons for the advice given	56	90.3%	67	91.2%	0.150	79	94.0%	72	83.7%	0.711	274	87.0%
6.Some things about the consultation with the physiotherapist or podiatrist could have been better (R)	46	74.2%	53	63.0%	0.166	68	72.3%	60	69.8%	0.120	227	72.1%
7.The physiotherapist or podiatrist listened very carefully to what I had to say	57	91.2%	68	93.2%	0.344	79	94.0%	74	86.0%	0.330	278	88.3%
8.I understand my treatment much better after seeing the physiotherapist or podiatrist	54	87.1%	54	74.0%	**0.025**	68	72.3%	61	70.9%	0.164	237	75.2%
9.The physiotherapist or podiatrist was interested in me as a person not just my illness	50	80.1%	56	76.7%	**0.033**	77	81.9%	65	75.6%	0.152	248	78.7%
10.I am NOT completely satisfied with the advice received from this physiotherapist or podiatrist (R)	56	90.3%	61	83.6%	**0.019**	75	79.8%	67	78.0%	0.455	249	79.0%
11.It was easy to make an appointment with the physiotherapist or podiatrist	35	56.5%	49	67.1%	0.900	74	78.7%	60	69.8%	**0.028**	218	69.2%
12.There was an acceptable time lapse to obtain an appointment	30	48.4%	43	58.9%	0.759	67	71.3%	57	66.3%	0.378	197	62.5%
13.It was possible to obtain an appointment on a convenient day or hour	40	64.5%	49	67.1%	0.695	70	74.5%	62	72.1%	0.067	221	70.2%
14.I can contact someone in the service by phone for help or advice in case of problem	38	61.2%	47	64.4%	0.881	70	74.5%	56	65.1%	**0.020**	211	67.0%
15.In an emergency I can get a quick appointment/consultation at this service	19	30.6%	25	34.2%	0.177	60	63.8%	36	41.9%	**0.001**	140	44.4%
16.I saw the physiotherapist or podiatrist at the appointed time	42	67.7%	62	84.9%	0.111	74	78.7%	73	84.9%	0.952	251	79.7%
17.The waiting time was acceptable	45	72.5%	64	87.7%	0.088	80	85.1%	71	82.6%	0.494	260	82.5%

*p based on Mann Whitney U test using 5-point Likert Scale; for ease of interpretation, the table only displays for each item the number of patients who indicated a positive response (i.e. Strongly Agree/Agree or Strongly Disagree/Disagree for negatively paraphrased items (R)) – all corresponding percentages relate to the entire subgroup at the top of the column i.e. interpreting no response to the specific item as a lack of a positive response

With respect to service access, patients of podiatrist independent prescribers were more satisfied with ‘the ease of making an appointment’ and ‘the ability to contact the service by phone or in times of emergency’ (see Table 5) than patients of the non-prescribing podiatrists, with no notable difference evident in patients attending physiotherapist prescribers compared to patients of non-prescribing physiotherapists.

There was no effect on the remaining four items reporting on ease of access on the acceptability of: i) waiting time to obtain an appointment; ii) obtaining an appointment on a convenient day or hour; iii) waiting time or iv) seeing the physiotherapist or podiatrist at the appointed time between patients attending a physiotherapist or podiatrist independent prescriber when compared to those attending a non-prescribing physiotherapist or podiatrist.

Patients of a physiotherapist or podiatrist independent prescribers were more likely to receive medicines information or advice during the consultation (58 out of 146 (39.7%) vs 37 out of 151 non-prescribing physiotherapist or podiatrist patients (24.5%); *p* = 0.005), with varying levels of satisfaction reported (see Table 6). Compared to patients of non-prescribing physiotherapist or podiatrists, patients of physiotherapist or podiatrist independent prescribers were significantly more likely to: ‘be told when’ and ‘how often’ to take their medicine, ‘intend to take their medicines’ and ‘find it easier to follow the physiotherapists’ advice’ (*p* ≤ 0.05).

b) *Quality of life- EQ-5D-L*

**Table 6:** Patient views and experience of medicines management advice and information provided by physiotherapist or podiatrist

Patient views and experience of medicines management advice and information provided by physiotherapist or podiatrist		Physiotherapist Independent Prescriber (n = 27)	Physiotherapist Non-prescriber (n = 24)			Mann- Whitney U-test	Podiatrist Independent Prescriber (n = 31)	Podiatrist Non-prescriber (n = 13)			Mann- Whitney U-test	Total	
		Strongly Agree/Agree					Strongly Agree/Agree					Strongly Agree/Agree	
	N (excluding not applicable^a^)	n	%	n	%	p*	n	%	n	%	p*	n	%
1. The physiotherapist or podiatrist gave me time to clarify questions I may have had about my medicine	84	24	96.0%	19	86.4%	0.627	21	84.0%	11	91.7%	0.901	75	89.3%
2. The physiotherapist or podiatrist told me when to take my medicine	64	11	73.3%	6	40.0%	**0.030**	19	82.6%	9	81.8%	0.719	45	70.3%
3. The physiotherapist or podiatrist told me how often I should take my medicine	61	12	85.7%	5	35.6%	**0.002**	19	86.4%	9	81.8%	0.835	43	70.5%
4. The physiotherapist or podiatrist provided me with information on the purpose of my medicine	75	16	73.7%	14	70.0%	0.547	19	82.6%	11	84.6%	0.549	60	80.0%
5. The physiotherapist or podiatrist provided me with information on how to use my medicine	59	11	73.3%	5	45.5%	0.062	16	80.0%	10	91.0%	0.608	42	71.2%
6. I expect that it will be easy to follow the physiotherapist’s or podiatrist’s advice about my medicine	68	12	75.0%	10	66.7%	0.181	22	91.7%	11	84.6%	0.346	57	83.8%
7. The physiotherapist or podiatrist told me the name of my medicine	71	17	85.0%	9	60.0%	0.178	18	75.0%	9	75.0%	0.354	53	74.6%
**Patient views and experience of medicines management advice and information provided by physiotherapist or podiatrist**		**Physiotherapist Independent** **Prescriber**		**Physiotherapist** **Non-prescriber**			**Podiatrist** **Independent Prescriber**		**Podiatrist** **Non-prescriber**			**Total**	
		**Strongly Agree/Agree**					**Strongly Agree/Agree**					**Strongly Agree/Agree**	
	**N** (excluding not applicable^a^)	**n**	**%**	**n**	**%**	p*	**n**	**%**	**n**	**%**	p*	**n**	**%**
8. The physiotherapist or podiatrist explained the side effects of my medicine	63	11	68.8%	12	70.6%	0.578	13	59.1%	5	50.0%	0.443	41	65.0%
9. I would have liked to have received more information about my medicine from the physiotherapist or podiatrist #	73	3	13.6%	3	17.6%	0.438	0	0.0%	3	25.0%	0.288	9	12.3%
10. The physiotherapist or podiatrist provided me with information on what to do if I missed a dose of my medicine	48	3	25.0%	3	27.3%	0.795	3	21.4%	1	9.1%	0.274	10	20.8%
11. It may be difficult for me to do exactly what the physiotherapist or podiatrist told me to do in relation to my medicine #	56	0	0.0%	1	9.1%	**0.038**	5	23.8%	1	9.1%	0.832	7	12.5%
12. I’m not sure it will be worth the trouble to take the medicine advised by the physiotherapist or podiatrist #	62	2	13.3%	1	8.3%	0.298	1	6.7%	1	8.3%	0.570	5	8.1%
13. Receiving a prescription for medicine from my physiotherapist or podiatrist reduced my waiting time today	40	4	30.8%	1	16.6%	0.919	6	46.1%	6	75.0%	0.446	17	42.5%
14. I am likely to take the medicine prescribed for me today	47	7	36.8%	2	28.5%	**0.022**	13	72.2%	11	100.0%	0.204	33	70.2%

^a^ those patients who did not respond “Yes” to the preceding question “During the consultation today, did the physiotherapist or podiatrist prescribe and/or give you advice and information about medicines(s)?”

*p-value based on Mann Whitney U test utilising the original 5 point Likert scale; for ease of interpretation, the table only displays for each item the number of patients who responded Strongly Agree/Agree

Note that for items labelled # this may not be regarded as a positive response

Indications at baseline were that patients who saw physiotherapist independent prescribers had lower generic quality of life than those seeing the non-prescribing physiotherapists, due to lower scores on the mobility dimension. However, there was no statistically significant difference between physiotherapist or podiatrist independent prescribers and non-prescribing physiotherapist or podiatrist groups on either individual items or overall EQ-5D-5Lscore (*p* ≥ 0.05) (Table 7, individual dimension scores not shown).

**Table 7:** Overall EQ. 5D index score: baseline and follow-up

	From the 129 completers	Baseline for 116 with EQ. 5D in BOTH data sets only	Follow-Up for 116 with EQ. 5D in BOTH data sets only		
	Number of patients completing BOTH sets of EQ. 5D questions	EQ 5D-5L Mean (SD)	EQ 5D-5L Mean (SD)	Change from Baseline (95% CI)^a^	Paired t-test p-value
PT IP	25	0.56 (0.31)	0.64 (0.27)	0.08 (−0.04 to 0.19)	0.194
PT NP	28	0.73 (0.19)	0.73 (0.22)	0.001 (−0.07 to 0.07)	0.973
PO IP	33	0.70 (0.26)	0.78 (0.20)	0.08 (0.003 to 0.16)	0.042
PO NP	30	0.66 (0.26)	0.76 (0.28)	0.10 (0.03 to 0.16)	0.004
All IP	58	0.64 (0.29)	0.72 (0.24)	0.08 (0.01 to 0.14)	0.019
All NP	58	0.69 (0.23)	0.75 (0.25)	0.05 (0.003 to 0.10)	0.036
All PT	53	0.65 (0.26)	0.69 (0.25)	0.04 (−0.03 to 0.10)	0.266
All PO	63	0.68 (0.26)	0.77 (0.24)	0.09 (0.04 to 0.14)	0.001

^a^[Positive change indicates mean improvement in health at Follow-Up]

Quality of life overall scores in both physiotherapist and podiatrist independent prescribers and non-prescribing groups improved significantly between baseline and follow-up. Differences in change scores between the physiotherapist and podiatrist independent prescribers and non-prescribing physiotherapist or podiatrists, however, were not statistically significant (Table 7). The sample for which data at both time points were available was limited (*n* = 116).

iv) *iv. Economic analysis*

Amongst physiotherapists, the independent prescribers had significantly longer consultation duration than non-prescribers (27.6 vs 20.8 min) (Table 8). Amongst podiatrists, the frequency with which medications i.e. a new medication, repeat medication (same dosage), or repeat medication (dosage changed) and tests were ordered were significantly higher in independent prescribers than non-prescribers (Table 8). There was a trend for consultation duration to be longer for independent prescribers (23.4 vs 19.9 min) (Table 8).

**Table 8:** Comparison of independent prescribers and non-prescribers, by profession, on variables used in the cost analysis

Professional group	Prescribing status		Number of medications required (Observation Q6)	Number of tests requested / patient (Sample audit)	Consultation time in minutes / patient (Observation Q1)	Discussions with colleagues in minutes/ per patient (Observation Q9,10)	x	Patients receiving referral (not for tests) (Observation Q11)	Patients with planned follow up (Observation Q15)	Patients reporting verified unplanned consultations within 2 months (Patient questionnaire)
**PHYSIO-** **THERAPY**	**Independent prescriber (IP)**	N	107	42	107	107	**N**	107	107	47
		Missing	9	74	9	9	**Yes N**	32	54 (8 by phone)	1
		N, % of zeros	75, 70.1%	32, 76.2%	0	88, 82.2%	**Yes %**	29.9%	50.5%	2.1%
		Mean	0.327	0.262	27.64	1.802				
		SD	0.546	0.497	14.10	5.585				
		Median	0	0	24	0				
		IQR	0 to 1	0 to 0.25	18 to 34	0 to 0				
	**Non prescriber (NP)**	N	115	44	115	115	**N**	115	115	46
		Missing	7	78	7	7	**Yes N**	34	51 (1 by phone)	2
		N, % of zeros	87, 75.7%	33, 75.0%	0	114, 99.1%	**Yes %**	29.6%	44.3%	4.3%
		Mean	0.252	0.250	20.83	0~				
		SD	0.456	0.438	10.46	0~				
		Median	0	0	19	0				
		IQR	0 to 0	0 to 0.75	14 to 28	0 to 0				
	**Significant difference (p)**		MWU 0.336	MWU 0.949	**MWU < 0.0005**	**MWU < 0.0005**		Chi Sq 0.956	Chi Sq 0.361	FE 0.617
**PODIATRY**	**Independent prescriber (IP)**	N	128	24	128	128	**N**	128	128	57
		Missing	5	109	5	5	**Yes N**	17	110 (0 by phone)	0
		N, % of zeros	93, 72.7%	17, 70.8%	0	109, 85.2%	**Yes %**	13.3%	85.9%	0%
		Mean	0.328	0.375	24.27	0.976				
		SD	0.616	0.647	24.32	2.682				
		Median	0	0	16	0				
		IQR	0 to 1	0 to 1	11 to 27.75	0 to 0				
	**Non prescriber (NP)**	N	124	32	123	124	**N**	124	124	47
		Missing	3	95	4	3	**Yes N**	6	111 (7 by phone)	1
		N, % of zeros	114, 91.9%	32, 100%	0	111, 89.5%	**Yes %**	4.8%	89.5%	2.1%
		Mean	0.105	0	16.88	0.726				
		SD	0.379	0	9.86	2.867				
		Median	0	0	16	0				
		IQR	0 to 0	0 to 0	10 to 23	0 to 0				
	**Significant difference (p)**		**MWU 0.001**	**MWU < 0.0005**	MWU 0.073	MWU 0.349		Chi Sq 0.20	Chi Sq 0.387	FE 0.452

Number of medications required = new medication + repeat medication (same dosage) + repeat medication (dosage changed)

Comparing physiotherapists and podiatrists, planning of follow up consultations was higher by podiatrist independent prescribers than physiotherapist independent prescribers, but no significant differences were found between independent prescribers and non-prescribers within the professions. After removing unplanned consultations in the two months after the original consultation that were considered (by two independent reviewers) to be unamenable to treatment delivered in the index consultation, only four items of unplanned service utilisation remained across the whole sample of patients of physiotherapists and podiatrists, all of which were related to pain relief (Table 8).

#### Costs of consultations

Difference in costs of consultation duration of independent prescribers compared to non-prescribers for physiotherapist and podiatrist groups were based on Agenda for Change (AfC) band 8a, which was the most frequent grade of physiotherapist and podiatrist independent prescribers in the study, i.e. £70 per hour [73]. Compared to the cost of a non-prescriber consultation, the independent prescriber consultation was, on average, more costly by £7.95 for physiotherapists (£24.30 vs £32.25) and £8.62 (£19.69 vs £28.31) for podiatrists. The salary of a grade 9 professional is twice that of grade 8a, so at that higher level, the differences in the cost of consultations between independent prescribers and non-prescribers would be doubled. Use of grade 7 instead of grade 8a would reduce the differences between independent prescribers and non-prescriber by about £1.20 per consultation. Amongst the podiatrists, the independent prescribers were at band 7 (advanced / team leader), 8a (principal) and 9 (consultant); two of the non-prescribers were band 9 and the third was band 6 (specialist). Participating physiotherapists were all band 8a, except one non-prescriber (grade 8c), and one independent prescriber (grade 7).

Costs could not be estimated for the other elements of activity that might differ between independent prescribers and non-prescribers due to data availability problems. Information on tests ordered were drawn from a small sample of records (n = max 15 per site) in each site (the audit); reporting of the type and dose of new medications, referrals and frequency of planned follow up was incomplete.

#### Discussions with colleagues

The independent prescribers in the physiotherapist group consulted colleagues about patients significantly more often than the non-prescribers (17.8% vs 0.9% of consultations), and most discussions were with medical colleagues, averaging 9.5 min per discussion (Table 9).

**Table 9:** Discussion with colleagues about patient

Professional group	Prescribing status	Number and % of all patients seen for whom discussion occurred with colleague	Mean (SD) minutes in discussions with colleague per patient	Discussion with same professional n, mean (SD) minutes	Same colleague cost / discussion^b^ (£, 2015)	Discussion with medical professional n, mean (SD) minutes	Medical colleague cost / discussion^b^ (£, 2015)
PHYSIOTHERAPY	Independent prescriber	19 (17.8%)	10.61 (9.68)	3, 19.5 (14.8)	£22.75	16, 9.5 (8.9)	£21.69
	Non prescriber	1 (0.9%)	0 (n/a)	1, time missing	Not known	0, n/a	0
	Significant difference	*p* < 0.0005^a^	n/a				
PODIATRY	Independent prescriber	19 (14.8%)	6.89 (3.20)	11, 6.8 (3.6)	£7.93	8, 7.0 (2.8)	£15.98
	Non prescriber	13 (10.5%)	6.92 (6.14)	12, 7.3 (6.3)	£8.52	1, 3.0 (0.0)	£6.85
	Significant difference	*p* = 0.299~	*p* = 0.493^				

^a^ Fishers Exact test; ~ Chi squared test; ^ Mann Whitney U test

^b^Unit costs of health and social care 2015 (Curtis and Burns 2015), pro rata based on £70/ h for same professional i.e. AfC band 8a, as in Ec2 above, and £137/ h for medical consultant

Podiatrists held discussions with colleagues for > 10% of consultations (14.8% IPs, 10.5% NPs, (Table 9)), for around 7 min. Independent prescribers discussed a higher proportion of patients with medical colleagues, than a colleague from the same profession, thereby likely to be incurring higher costs. However, information on colleagues consulted was not precise, so calculations were indicative only. Some podiatrists were band 9 (consultant), so reporting discussions with ‘same’ professional would imply higher costs than are indicated in the table, which are based on AfC band 8a.

#### Cost implications

The available data suggest that for both physiotherapists and podiatrists in this study, care delivery by independent prescribers is more resource intensive and costly than non-prescribers due to longer consultations for physiotherapists and taking more time of colleagues to discuss patients. Whilst not costed, podiatrist independent prescribers had higher frequency of ordering medications and tests than non-prescribing podiatrists. Analysis of the changes in self-reported health status between baseline and 2 months follow up using EQ-5D-5L found no difference in change scores of independent prescribers and non-prescribers for either physiotherapists or podiatrists, but these data were only available for a small sample of participants.

## Discussion

This is the only known national evaluation of physiotherapist and podiatrist independent prescribers in the UK or the world, and the first to adopt a comparative case study design to compare outcomes and costs for patients managed by physiotherapist and podiatrist independent prescribers/ non-prescribers. Unlike nurses and pharmacists, where prescribing has been explored in some detail using self-reported outcomes [26, 47, 70], there is a dearth of equivalent information in the allied health professions, including either physiotherapy and/ or podiatry [29, 35] and/ or studies adopting direct observation of outcomes [26]. Our study demonstrates that care provided by physiotherapist and podiatrist independent prescribers is equivalent, in terms of quality of life and patient satisfaction, to care provided by non-prescribing physiotherapists with prescribing undertaken by doctors. Independent prescribing by physiotherapists and podiatrists was found to be effective, and highly acceptable, with higher levels of patient satisfaction in some aspects of medicines information also reported than for non- prescribers.

Importantly, it appears that physiotherapist and podiatrist independent prescribing is developing in line with original policy intention to improve access to medicines and quality of care across a range of settings [74–76]. The evidence generated in this study demonstrates that physiotherapist and podiatrist independent prescribers can provide a high standard of care. Extending non-medical staff, such as physiotherapists’ and podiatrists’, scope of practice to include independent prescribing is key to supporting effective delivery of the NHS Long Term Plan [9, 49, 77], and creating a step change in developing the capacity and capability of the workforce to deliver innovative models of service delivery [4, 9]. The severity of the workforce deficit makes changes, such as the increased level of clinical autonomy, associated with independent prescribing an attractive option to commissioners who seek to address gaps in service delivery. As the world leader in extending prescribing rights to nurses, pharmacists and allied health professions the findings are of significant importance to international policy makers who seek to learn from the pioneering advancement of prescribing rights in UK [25, 28] to inform their own approach to addressing the workforce deficit.

Internationally it is now common for physiotherapists, nurse practitioners, pharmacists, social workers, and psychiatric nurses to be located within extended primary care teams [78] with plans to extend this further recently announced [9, 11]. Nearly 50% of appointments in UK general practice are for example, already provided by non-medical staff, i.e. nurses, pharmacists and allied health professionals [9, 79]. In addition to the current shortage of 2500 general practitioners, this is important for several reasons: i) the current deficit in primary care looks set to continue [80, 81]; ii) the recent proposal for home visits to be removed from the GP contract, and iii) the government pledge to create 50 million more GP appointments year by 2024/25 [81, 82]. As the third largest workforce in health and care in England, allied health professionals have, through the introduction of a further 20,000 non-doctor roles in primary care [83], great potential to contribute to transforming care and ensuring ongoing access to medicines [11].

Having a robust economic evaluation of physiotherapist and podiatrist independent prescribing is particularly important, given that identifying a sustainable solution that i) improves the worldwide deficit of health workers, and ii) makes best use of limited resources is essential to ensuring ongoing access to medicines [9, 11]. Our cost appraisal from the case sites suggests that physiotherapist and podiatrist independent prescriber care delivery is more resource intensive than non-prescribing physiotherapists and podiatrists. This arises through longer consultation duration, more ordering of medicines and tests (podiatrists) and more discussions with colleagues (physiotherapists). These costs, however, need to be considered in relation to benefits, particularly clinical outcomes, many of which could not be measured in this study. Only a limited economic analysis was possible meaning that the findings should be treated with caution. Whilst the original intention had been to undertake a patient level micro costing analysis, data deficiencies limited what could be included. Further research is required to understand how team configuration affects care delivery, patient outcomes and costs.

The most complete data were available for consultation duration, and the calculation of associated costs showed independent prescribers to incur slightly higher consultation costs than non-prescribers in both the podiatrist and physiotherapist groups (£8.62 and £7.95 respectively). It is important to note however that consultation duration and associated costs may simply be driven by professional differences and clinic practices. The complexity of these arrangements means that the differences in cost could equally reflect service differences which would exist regardless of independent prescribing status. Furthermore, the time spent in discussion with colleagues may reflect the multi-professional service that many case sites provided. Multi-professional, or team-working is a fundamental component of health care delivery in the UK and central to current government policy [84–86]. There is increasing emphasis on establishing systems, rather than single episodes of care, that dissolve traditional boundaries [87, 88] to support the increasing number of people with long-term conditions.

There is limited evidence available with which to compare our study findings [26, 35, 45, 46, 89]. Despite positive findings that non-medical prescribing is safe, and provides beneficial clinical outcomes [26, 28, 70], the impact on the health economy, as reported in two recent systematic reviews examining clinical and cost effectiveness, remains unclear [45, 46, 89]. The authors, as in this study, highlight the difficulty in separating non-medical prescribing effects from the contributions of healthcare team members, and a lack of adequately powered randomised controlled trials examining non-medical prescribing across clinical specialities, professions and settings [25, 45]. Given that extended prescribing rights to nurses, pharmacists and allied health professions offers a sustainable approach to improving the global workforce deficit, there is a pressing need to establish economic benefits, or otherwise of non-medical prescribing to inform future international policy developments. A different approach, involving highly targeted specific outcomes, and or longitudinal studies is therefore required. The development of a minimum data set of important outcome measures for non-medical prescriber assessment would as Noblet et al. suggests [45], be highly beneficial, and generate the required evidence to evaluate the overall benefit of non-medical prescribing and inform future developments in the UK and around the world.

### Strengths and limitations

In the first study to explore allied health professional prescribing, the 14 case sites supported an in-depth evaluation and comparison of physiotherapist and podiatrist independent prescribers to non-prescribers in a range of care settings. Use of multiple methods of data collection, including an observational component, strengthens the trustworthiness of the findings. Physiotherapist and podiatrist independent prescriber participants were selected from a larger sample (*n* = 70) who completed a trainee physiotherapist and podiatrist independent prescriber survey and indicated that they would be willing to be involved in further research [52].

Despite challenges in matching sites, given the diversity of service settings, roles, and patient needs, between and within the two professions, patient characteristics indicated good matching on most factors. For future research, matching at a patient/condition level would ensure a comparative sample. Additionally, as patients were predominantly retired, house owners, and lacked ethnic diversity, reflecting study locations, caution must be applied with respect to generalizing the findings to other groups of the population. Furthermore, there are limitations and methodological challenges associated with using the same evaluation measures on two different professional groups for whom separate measures might have been more appropriate. The economic analysis was constrained as described above. An analysis of effectiveness was not possible because it was not feasible to collect data on specific indicators for change across the wide variety of conditions treated within physiotherapist and podiatrist consultations. Our ability to link each of the various aspect of patient data (i.e. observation, questionnaires, record audit) was also very limited as patients, in line with good ethical practice, had the option to select which aspects of data collection they agreed to. As a result, it was not possible to match patients across the different data sets, or to complete some of the intended analysis.

## Conclusions

This study provides new knowledge about physiotherapist and podiatrist independent prescribing, the high level of care and patient satisfaction they provide. Given that extending prescribing responsibilities to nurses, pharmacists, and allied health professionals is increasingly being recognised as effective way to alleviate shortfalls in the global health workforce and ensure ongoing access to prescription medicines around the world this is important. PP-IP care delivery was found to be more resource intensive than NP-PP. However, this study is limited, and findings needs to be verified through further research, including a full economic analysis. A more focussed longitudinal exploration within each profession with targeted outcome measures would enable a more robust comparison of the impact of physiotherapist and podiatrist independent prescribing across the United Kingdom and inform further developments around the world.

## Data Availability

The study did not receive ethics approval, or participant consent, to place a study dataset in the public domain. The data, and tools used for its collection in this study can be made accessible to qualified researchers upon reasonable request pursuant to any restrictions required to ensure the privacy of human subjects involved. Access to data will be subject to a data sharing agreement approved by University of Surrey. Researchers interested in accessing USEFUL data should send their request to the Director of Research, Professor Emma Ream (e.ream @surrey.ac.uk).

## Abbreviations

A&E: Accident and Emergency
BP: Blood pressure
BNF: British National Formulary
CT: Computerised tomography
EQ 5-D: EuroQol 5-D
GP: General Practitioner
MMA: Medicines Management Activities
MRI: Magnetic Resonance Imaging
NMP: Non- medical prescriber
NP-PP: NON-prescribing Physiotherapist & Podiatrist
OTC: Over the counter
PGD: Patient Group Direction
PO-IP: Podiatrist independent prescriber
PO-NP: Podiatrist non-prescriber
PP: Physiotherapist & Podiatrist
PP-IP: Physiotherapist & Podiatrist Independent Prescriber
PSD: Patient Specific Direction
PT-IP: Physiotherapist independent prescriber
PT-NP: Physiotherapist non-prescriber
